# Down Syndrome Cognitive Phenotypes Modeled in Mice Trisomic for All HSA 21 Homologues

**DOI:** 10.1371/journal.pone.0134861

**Published:** 2015-07-31

**Authors:** Pavel V. Belichenko, Alexander M. Kleschevnikov, Ann Becker, Grant E. Wagner, Larisa V. Lysenko, Y. Eugene Yu, William C. Mobley

**Affiliations:** 1 Department of Neurosciences, School of Medicine, University of California San Diego, La Jolla, CA, 92093–0649, United States of America; 2 Genetics Program and Department of Cancer Genetics, Roswell Park Cancer Institute, Buffalo, NY, 14263, United States of America; IGBMC/ICS, FRANCE

## Abstract

Down syndrome (DS), trisomy for chromosome 21, is the most common genetic cause of intellectual disability. The genomic regions on human chromosome 21 (HSA21) are syntenically conserved with regions on mouse chromosomes 10, 16, and 17 (Mmu10, Mmu16, and Mmu17). Recently, we created a genetic model of DS which carries engineered duplications of all three mouse syntenic regions homologous to HSA21. This ‘triple trisomic’ or TTS model thus represents the most complete and accurate murine model currently available for experimental studies of genotype-phenotype relationships in DS. Here we extended our initial studies of TTS mice. Locomotor activity, stereotypic and repetitive behavior, anxiety, working memory, long-term memory, and synaptic plasticity in the dentate gyrus were examined in the TTS and wild-type (WT) control mice. Changes in locomotor activity were most remarkable for a significant increase in ambulatory time and a reduction in average velocity of TTS mice. No changes were detected in repetitive and stereotypic behavior and in measures of anxiety. Working memory showed no changes when tested in Y-maze, but deficiency in a more challenging T-maze test was detected. Furthermore, long-term object recognition memory was significantly reduced in the TTS mice. These changes were accompanied by deficient long-term potentiation in the dentate gyrus, which was restored to the WT levels following blockade of GABAA receptors with picrotoxin (100 μM). TTS mice thus demonstrated a number of phenotypes characteristic of DS and may serve as a new standard by which to evaluate and direct findings in other less complete models of DS.

## Introduction

**Fig 1:**
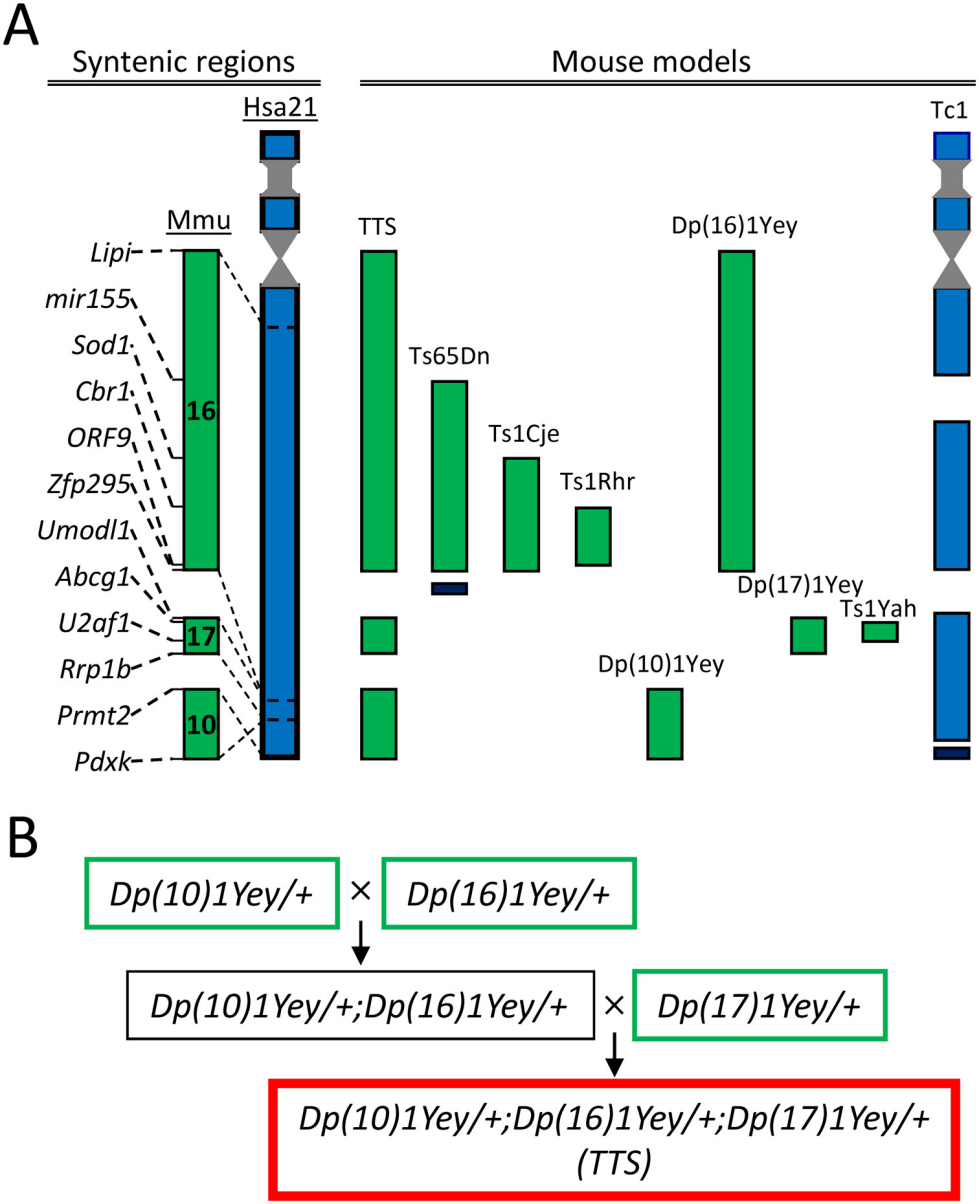
Mouse genetic models of DS and breeding schema of TTS mice. **A:** Mouse genetic models. *Left*: Correspondence of syntenic genomic regions on human chromosome 21 (HSA21) to mouse chromosomes (Mmu) 10, 16, and 17. *Right*: Schema of the triplicated genomic regions in several notable mouse models of DS. Mouse and human genomic regions are shown in green and blue respectively. Triplicated genomic regions not present on HSA21 and, therefore, not triplicated in DS, are shown in black. See Table 1 for details regarding the size and boundaries of the triplicated regions. **B:** Breeding schema of TTS mice. First, Dp(10)1Yey/+, Dp(16)1Yey/+, and Dp(17)1Yey/+ mice (green rectangles) were generated by triplications of the corresponding syntenic regions. Next, compound Dp(10)1Yey/+;Dp(16)1Yey/+ mice (black rectangle) were generated by crossing Dp(10)1Yey/+ and Dp(16)1Yey/+ mutant mice. Finally, TTS mice (red rectangle) were generated by crossing Dp(10)1Yey/+;Dp(16)1Yey/+ and Dp(17)1Yey/+ mice.

Down syndrome (DS; Trisomy 21) is caused by the presence within the genome of a third copy of human chromosome 21 (HSA21) [1]. A number of notable mouse genetic models have been created to assess genotype-phenotype relationship in DS [2,3,4,5,6,7,8,9,10,11,12,13]. In the mouse, the genomic regions orthologous to HSA21 are located on chromosomes 10, 16, and 17 (Mmu10, Mmu16, and Mmu17). The largest genetic region, including the ‘Down syndrome critical region’ (DSCR), is located on Mmu16 (Fig 1A). Models of DS that feature a third copy of all or part of Mmu16 (e.g., Ts16, Ts65Dn, Ts1Cje, Ms1Cje, Ts1Rhr, Ts1Yah, etc) have provided important insights into the genetic and mechanistic bases of many DS phenotypes [14,15,16,17,18]. Notably, the Ts65Dn mouse, which is segmentally trisomic for the Mmu16 region from mir155 to Zfp295 (~100 genes), has made it possible to explore a number of behavioral, physiological, and other neurobiological differences characteristic of DS. For example, Ts65Dn mice showed deficient performance in the Morris water maze [19,20,21,22,23,24], contextual fear conditioning [25,26,27,28], novel object recognition [23,26,29,30], other memory tests [31,32,33], as well as deficient long-term potentiation (LTP) in the CA1 [34,35,36,37], dentate gyrus (DG) [26,30,38], and striatum [39], as well as age-related degeneration of neuronal populations characteristically impacted by DS and AD [9,40,41].

Ideally, mouse studies of DS phenotypes would build on findings in a model containing all of the mouse homologues of HSA21 genes. Admitting the structural and genetic differences between HSA21 and its murine orthologs and the possibility that such differences may obviate attempts to replicate all DS phenotypes, studies of such a mouse model would serve as a kind of ‘gold standard’ by which to evaluate and direct findings in less complete mouse trisomic models. Recently, advances in chromosomal engineering were used to create such mouse model [42]. This model of DS carries an extra copy of all three segments syntenic to HSA21: Mmu10 (from Pdxk to Prmt2, 41 genes), Mmu16 (from Lipi to Zfp295, 115 genes), and Mmu17 (from Umodl1 to Rrp1b, 19 genes). Bearing initially the name ‘Dp(10)1Yey/+;Dp(16)1Yey/+;Dp(17)1Yey/+’ [42], herein these mice we will refer to as the triple trisomic (TTS) model of DS. Complementing an earlier report [42], here we considerably extended the analysis of behavior and cognition in TTS mice and examined synaptic plasticity in the dentate gyrus. We show that TTS mice demonstrate deficiencies in each of these domains. Thus, TTS mice can serve to define what DS phenotypes can be recapitulated in mice and to guide the additional studies needed to define and explore phenotype-genotype relationships in DS.

## Materials and Methods

### Animals

As detailed previously [10,42], Dp(16)1Yey/+ (i.e. Ts1Yey), Dp(10)1Yey/+ (i.e., Ts2Yey), and Dp(17)1Yey/+ (i.e., Ts3Yey) mice were first generated by triplications of syntenic regions orthologous to their counterparts on HSA21 (see Table 1). The individual duplications in Dp(16)1Yey/+, Dp(10)1Yey/+, and Dp(17)1Yey/+ mice were identified by Southern blot analysis. After colonies of these mice were established, Dp(16)1Yey/+, Dp(10)1Yey/+, and Dp(17)1Yey/+ were backcrossed to wild-type C57BL/6J mice for five generations and a standard breeding strategy was used to generate compound mutant mice carrying all three duplications, thus generating Dp(10)1Yey/+;Dp(16)1Yey/+;Dp(17)1Yey/+ (i.e. Ts1Yey;Ts2Yey;Ts3Yey) or triple-trisomic (TTS) mice (Fig 1B). Their normosomic ‘wild type’ (WT) littermates were used as controls. The genotype was confirmed by Agilent microarray-based comparative genomic hybridization as described previously [43,44]. Male adult mice with the age of 4–8 months were used in all experiments. Mice were housed 2 to 4 per cage with a 12 h light-dark cycle and ad lib access to food and water. It is noteworthy that producing the TTS mouse includes multiple crossings and, therefore, necessitates significant effort and cost. The Mendelian ratio predicts only 12.5% of the progeny from the last crossing carry the triple-trisomies, and the real percentage is significantly lower due to DS-associated abnormalities [43]. For this reason, although all available mice were used in experiments, the numbers of TTS and WT mice (TTS = 12; WT = 22), while fully adequate, were relatively small and the age range included was somewhat larger.

**Table 1:** Major behavioral and physiological phenotypes in mouse genetic models of Down syndrome.

	TTS	Ts65Dn	Ts1Cje	Ts1Rhr	Dp(10)1Yey	Dp(16)1Yey	Dp(17)1Yey	Ts1Yah	Tc1
**The first reference**	[43]	[54]	[17]	[18]	[42]	[42]	[42]	[16]	[15]
**Added genetic regions /# of genes/**									
Mmu10	Pdxk-Prmt2/41 genes/				Pdxk-Prmt2/41 genes/				
Mmu16	Lipi-Zfp295, /115 genes/	mir155-Zfp295 /100 genes/	Sod1- Zfp295/69 genes/	Cbr1-ORF9/29 genes/		Lipi-Zfp295, /115 genes/		Abcg1–U2af1 /12 genes/	
Mmu17	Umodl1-Rrp1b /19 genes/						Umodl1-Rrp1b /19 genes/		
Hsa21									~92% of all Hsa21 genes
**Body weight**	↓[43]☑	↓ [55,56]	↔ [3]	↔[45,57]	─	─	─	─	─
**Physical fitness**									
Grip force	↓ [43]	↓ [58]	─	─	─	─	─	─	↔ [59]
Ambulatory velocity	↓ ☑	↑[25,26,37]	─	↔ [45]	─	─	─	─	↑ [59]
Swimming velocity	↓ [43]	↓[58,60];↔[24]	─	─	↔ [42]	↓[42]	↔ [42]	↔ [16]	─
**Locomotor activity**									
Ambulatory distance	↔ ☑	↑[25,26,37]	─	↔ [45]	─	─	─	↔ [16]	↑ [59]
Ambulatory time	↑ ☑	↑[26,37]	─	─	─	─	─	─	─
Jumps/Jumping time	↓ ☑	↑[25]	─	─	─	─	─	─	─
Vertical time/counts	↔ ☑	↑ [25]	─	↓ [45]	─	─	─	─	─
Other activity tests			↓[17]						
**Stereotypic and compulsive behavior**									
Stereotypic	↔ ☑	↑[61,62]	─	─	─	─	─	─	↑ [59]
Compulsive	↓ ☑	─	─	─	─	─	─	─	─
**Anxiety**									
Thigmotactic behav.	↔ ☑	↔[26]; ↑[37]	─	↑ [45]	─	─	─	↔ [16]	↓ [59]
Pellet test	↔ ☑	↔[26]	─	↔ [45]	─	─	─	─	─
Other anxiety tests				↔[63]				↔ [16]	↔ [15]
**Working memory**									
Y-maze	↔ ☑	↓ [25,37]	─	─	─	─	─	↓ [16]	─
T-maze	↓ ☑	↓[25,26]	↓ [3]	↓ [45]	─	─	─	─	↔ [15]
**Long-term memory**									
Morris water maze	↓ [43]	↓[19,20,21,22,23,24]	↓[17,64]	↔[21]	↔[42]	↓[42]	↔ [42]	↑ [16]	↔ [65]
CFC	↓ [43]	↓ [25,27,28]	─	─	↔[42]	↓[42]	↔ [42]	─	─
NOR	↓ ☑	↓ [23,25,26,37,66]	↔ [67]	↓ [45]	─	─	─	─	↔ [65]
**Synaptic plasticity**									
CA1 HFS	─	↓[34];↔[35]	↓[68]	↔[21]	─	─	─	↑ [16]	─
CA1 TBS	↓[43]	↓ [35,36,37]	─	─	↔[42]	↓[42]	↑[42]	─	─
CA1 TBS+GABAA ant	─	↔ [35]	─	─	─	─	─	─	─
DG HFS	↓ ☑	↓[26,30,38]	↓ [3]	↓ [45]	─	─	─	─	↓ [15,65]
DG HFS+GABAA ant.	↔ ☑	↔ [30,38]	↔ [3]	↔ [45]	─	─	─	─	─

‘☑’–Results of this study;

‘↔’—No change;

‘↑’–Increased;

‘↓’—Reduced;

‘─’–Data not published.

The study was conducted in accordance with the National Institutes of Health guidelines for the care and use of animals. All experiments in this study were performed according to approved protocols from the Roswell Park Cancer Institute and the University of California San Diego (UCSD) Institutional Animal Care and Use Committees, and all efforts were made to minimize animal stress and discomfort.

### Behavioral tests

The mice were behaviorally tested as a single cohort at the age of 4–8 months as described [45,46]. Briefly, each mouse was handled for 5 minutes, twice a day, during the 7 days preceding testing and for a minimum of 3 days long rest period between tests. The tests were administered in the following order: locomotor activity, Y-maze, glass marble test, novel object recognition test, and T-maze test. All behavioral tests were performed during the light cycle between 10:00 am and 3:00 pm. On the day of testing, mice were transferred into the testing room in their home cages and allowed to accommodate for 2 hours. To minimize olfactory cues, each apparatus was thoroughly cleaned with 10% ethanol after each animal. TTS and WT mice were tested in random order by an investigator blinded to the animal genotype.

### Spontaneous locomotor activity

Spontaneous locomotor activity was monitored in a single trial using activity chambers made of acrylic glass and equipped with three planes of infrared detectors (model MED-OFA-MS; Med Associates, St. Albans, Georgia) with dimensions 27 cm long × 27 cm wide × 20.3 cm high. Four mice were tested concurrently in individual chambers. The area of the chamber was divided virtually on center (20 cm x 20 cm, zone 1) and periphery (the rest of the chamber, zone 0). The activity chambers were located within sound-attenuating boxes (66 x 55.9 x 55.9 cm) with a built-in internal fan for background noise (65 dB) and lighting for ambient illumination. For testing, an animal was placed in the center of the testing arena under ambient light (40 lux) and allowed to move freely for 10 minutes. Activity monitor software (Activity Monitor, version 4.3.6, Med Associates, St. Albans, Georgia) was used for recording and analysis of activity, as previously described [45]. According to the manufacture’s manual, a movement was registered as stereotypic if the subject crossed the beams without leaving the square area with a size of ‘4 beams x 4 beams’ (6.4 cm x 6.4 cm), and a movement was registered as ambulatory if the subject left such area.

### Glass marble test

Marble burying behavior was assessed as was previously described [47,48]. The testing apparatus (33 cm long × 21 cm wide × 19 cm tall) consisted of a polycarbonate mouse cage filled to a depth of 5 cm with wood bedding. Prior to each test, 20 glass marbles of diameter 11 mm were evenly arranged in a grid-like fashion on the bedding surface. After placing a mouse into the apparatus with light intensity about 30 lux, the apparatus was covered with a transparent lid with air holes. After the completion of 20 min, the mouse was removed from the apparatus and the number of buried glass marbles was counted. A marble was considered buried if ≥ 50% of the marble was covered by bedding. The number of buried marbles (M) was then expressed as percent of the total according to the formula: R(%) = M*100/20.

### Y-maze

Y-maze testing was performed using an apparatus with three equal arms (30 cm long × 10 cm wide × 20 cm high), made of opaque acrylic. A mouse was placed at the maze center under ambient illumination (20 lux) and allowed to explore the environment for 5 min. An arm entry was scored when the mouse entered the arm with all four paws. The total number of entries (N) and the number of ‘correct’ triplets (M, consecutive choices of each of the three arms without re-entries) was evaluated. The alternation rate was computed according to the formula: R (%) = M*100/(N-2).

### T-maze

T-maze testing was performed using an automated computerized T-shaped apparatus made of opaque acrylic. The apparatus had a start arm (30 cm length, 10 cm width, and 20 cm height) and two goal arms (30 cm length, 10 cm width, and 20 cm height). Vertical guillotine-type doors were located at the start arm, 10 cm from the arm end, and at two goal arms, 1 cm from the arm’s entrances. The position of animal in the maze was monitored by a set of infrared emitter/sensor photodiode pairs located near the guillotine doors. Depending on the animal’s position in maze, the doors were either opened or closed by a computer program, thus restricting movement of the experimental animal. The apparatus was illuminated with light intensity about 18 lux. Two-trial protocol was used. An experiment started with placing a mouse inside the closed compartment of the start arm, with its back to the door. After an initial 10 sec delay, the doors of the start arm and one of the goal arms were lifted and the mouse was allowed to explore the maze (acquisition trial). After the mouse eventually entered the opened goal arm, which was detected through crossing of an infrared beam 1 cm behind the door, the arm door was closed for 10 s allowing the mouse for the arm exploration. After this period, the arm door was again opened and the mouse was allowed returning to the start arm. Following a pre-determined period of 2 s, all three doors were opened and the mouse was allowed to explore the entire maze (test trial). During the maze exploration, the mouse eventually chose either the previously visited arm or the opposite arm, which was scored as no alternation or an alternation respectively. After the mouse again returned to the start arm, all maze doors were closed for 30 sec to erase memory traces from previous trials. Such acquisition/test procedures were repeated 6 times, after which the mouse was returned to the home cage. The mice were tested in 2 consecutive days, and the average alternation rate was computed according to the formula: R (%) = M*100/N, where M—number of alternations; N—total number of test trials.

### Novel object recognition

Two sample objects in one environment were used to examine long-term memory in TTS and WT mice, as previously described [45,49]. Before testing, mice were habituated in a chamber (31 long×24 wide×20 cm high) made of black acrylic glass for 10 minutes on 2 consecutive days under ambient light conditions (30 lux). Object preference was examined in prior experiments with mice of similar age, and objects with equal preferences were randomly selected as ‘familiar’ and ‘new’. The activity of mice during the task was recorded with a video camera. First, two identical ‘familiar’ objects were placed in the chamber, and a mouse was placed at the mid-point of the wall opposite the sample objects. After allowing the mouse 10 minutes to explore the objects, it was returned to the home cage. After a 24 hours interval (the retention period), one of the ‘familiar’ objects used for the memory acquisition was replaced with a ‘new’ object similar to the ‘familiar’ one in volume and major dimensions. The positions (left or right) of the ‘new’ and the ‘familiar’ objects during the testing phase were counterbalanced between mice and sessions. The mouse was again placed in the chamber for 3 minutes to explore the objects; the time spent investigating the objects was assessed. The animal was considered to be exploring the object if its head was directed at the object and the distance between the object and the mouse nose was 1 cm or less. Results were averaged for total exploration time of each object. Animals with the total exploration time during testing less than 4 s (n = 2 for each WT and TTS groups) were excluded from the analysis. The discrimination ratio was evaluated according to the formula: R(%) = T_new_*100/(T_new_ + T_old_), where T_new_ and T_old_ are the time periods spent on investigating the novel and familiar objects, respectively.

### Electrophysiology

The mice used for the electrophysiological studies were age 4–5 months. Physiological recordings were performed as described [38,50]. The mice were anesthetized with isoflurane before decapitation. The brain was quickly removed and immersed for 2–3 minutes in ice-cold artificial cerebro-spinal fluid (ACSF), containing (in mM): 119 NaCl, 2.5 KCl, 2.5 CaCl2, 1.3 MgSO4, 1 NaH2PO4, 26 NaHCO3, and 10 glucose, osmolarity 310 mOsm; the medium was continuously bubbled with carbogen (mixture of 95% O2 and 5% CO2), pH 7.4. The hippocampus was extracted and cut in ice-cold ACSF with a vibratome (Leica 1000; Leica, Nussloch, Germany) into 350-μm-thick transverse slices, which were allowed to recover in oxygenated ACSF at 32°C for 30 minutes and then at room temperature for an additional 1–5 hrs before experimental recordings.

For recordings, a slice was transferred into the recording chamber and superfused with ACSF at a constant rate of 2.5 ml/min at a temperature of 32°C. In the experiments with suppression of inhibition, picrotoxin was applied in the perfusion solution at concentration of 100 μM. Recording electrodes were made of borosilicate glass capillaries (1B150F; World Precision Instruments, Sarasota, FL) filled with ACSF (resistance, 0.2–0.4 MΩ). The stimulating monopolar electrodes were made of Pt/Ir wire, diameter 25 μm (WPI Inc, Sarasota, FL). The stimulating and recording electrodes were inserted under visual control into the middle molecular layer of dentate gyrus at a distance 300–350 μm from each other. Testing stimuli evoked field excitatory postsynaptic potentials (fEPSPs) that were 70–75% of maximum. The magnitude of the fEPSP was measured as the initial slope of the linear part of the fEPSP (0.1–0.9 msec after the presynaptic volley). LTP was induced by tetanizations with four trains of stimuli (0.5 sec at 100 Hz) with an inter-train interval of 10 s.

### Statistical analyses

The data for body weight, behavioral testing, and neurophysiology were exported to Excel (Microsoft, Redmond, WA) and statistical comparisons were performed. First, all parameters were examined for normality of distributions and passed this test. Second, F-test was performed to assess for inequality of variances. Because most of behavioral measurements showed unequal variances for TTS and WT groups, which was expected on the basis of the difference in animal number in the TTS and WT groups, ‘T-test with the assumption of unequal variances’ was used. For electrophysiological data, two samples and two-tailed Student’s t-test was used. Third, to account for possible age effects, correlation coefficients between the animal age and the measured behavioral parameters were evaluated. Neither of these correlations reached a level of significance (S1 Table) suggesting no impact of the animal’s age on the measured parameters. All results are expressed as mean ± SEM and reported according to recommendations of the American Psychological Association (APA). P values < 0.05 were considered to be significant.

## Results

Adult male TTS and WT control mice were examined in a battery of behavioral tests including: (i) Activity box (spontaneous locomotion and thigmotactic behavior); (ii) Glass marble test (obsessive-compulsive behavior); (iii) Y-maze (working memory and locomotion); (iv) Novel object recognition with the retention period of 24 h (long-term memory); (v) T-maze (working memory and locomotion). A separate cohort of animals (n = 4 of each TTS and WT) was used in electrophysiological experiments to study synaptic plasticity in the dentate gyrus.

### Body weight

Body weight is a manifestation of systemic health. Reduced weight is characteristic in children with DS [51]. Adult individuals with DS often show increased body weight due to obesity [52]. We compared body weight at 4–6 and 6–8 months of age in TTS and WT mice. Body weight was significantly reduced in the TTS mice at both age intervals. Thus, at 4–6 months body weight was in WT: 36.8 ± 1.1, TTS: 30.7 ± 2.2, t (8) = 2.69, p = 0.01. At 6–8 months the body weight was in WT: 38.5 ± 1.1, TTS: 33.1 ±1.4, t (11) = 3.4, p = 0.004. In Table 1, we compare our findings to those for other mouse models of DS. Note that the reduction in body weight in the TTS mouse, as compared to WT controls, was recapitulated in some but not all models in which this phenotype was recorded.

### Spontaneous locomotor activity

**Fig 2:**
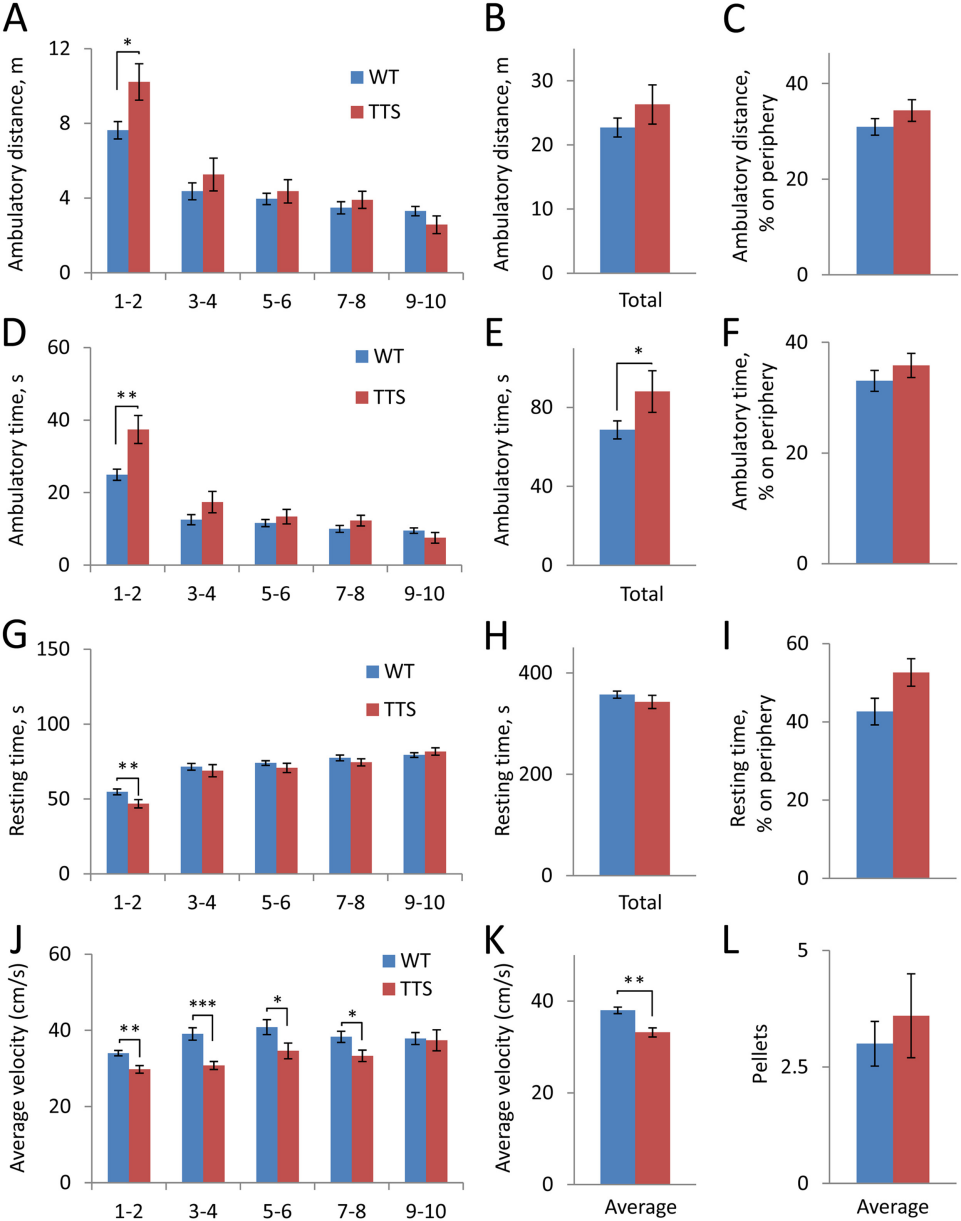
Spontaneous locomotor activity. A-C: Ambulatory distance; D-F: Ambulatory time; G-I: Resting time; J-K: Average velocity. The values averaged for 2-min sequential periods (A, D, G, J) and for the total 10-min testing period (B, E, H, K) are shown. Also, the percentage of distance and time on the arena periphery (C, F, I) are shown. L: Number of pellets dropped during the test. All results are mean ± SEM. Number of mice examined: WT = 22; TTS = 12. *p < 0.05, **p < 0.01, and ***p<0.001, significantly different from WT mice.

Profound perceptive-motor slowness and increased rates of the hyperkinetic disorders is characteristic of DS. We reasoned that, in animal’s models, these phenotypes might be reflected in altered parameters of locomotor activity. To assess spontaneous locomotor activity of TTS mice, we examined behavior in ‘activity box’ and measured such parameters as ambulatory distance (Fig 2A–2C), ambulatory time (Fig 2D–2F), resting time (Fig 2G–2I), and average velocity (Fig 2J and 2K). Locomotor activity was significantly increased in TTS vs. WT mice during the first 2-min testing period. This increase was registered as a significant increase in ambulatory distance (t(16) = 2.5, p = 0.02) (Fig 2A), ambulatory time (t(15) = 3.1, p = 0.003) (Fig 2D), and a decrease in resting time (t(22) = 2.4, p = 0.01) (Fig 2G). Ambulatory time averaged for the total 10-min testing period was also significantly greater in the TTS vs. WT mice (t(15) = 1.8, p = 0.049) (Fig 2E). Interestingly, the increase in locomotor activity was accompanied by a reduction in average velocity (t(29) = 3.7, p < 0.001) (Fig 2J and 2K). As a result, total ambulatory distance, which is a function of both time and velocity, did not differ significantly in TTS vs. WT mice (t(16) = 1.1, p = 0.14) (Fig 2B). Likewise, total resting time was also not different in TTS vs. WT mice (t(18) = 1.0, p = 0.16) (Fig 2H).

Thigmotactic behavior, which is characterized by clinging of animals to vertical walls, is often used as a measure of anxiety [53]. To examine thigmotactic behavior in TTS mice, we measured ambulatory distance, ambulatory time, and resting time at the arena center vs. arena periphery. Remarkably, none of these measures was different between TTS mice and their WT controls (Fig 2E–2G; p = 0.2–0.5) suggesting that TTS mice were not more anxious under the test conditions than WT controls. To assess anxiety in a different way, the number of fecal pellets released during the 10-min test was counted. Again, TTS and WT mice showed equal results (Fig 2H; t(18) = 0.63, p = 0.27). These results suggest that the levels of anxiety were equal in TTS and WT mice under the test conditions.

As shown in Table 1, the increase in locomotor activity was also seen in other models of DS, such as the Ts65Dn and Tc1 mice, while a decrease in ambulatory velocity in DS models has not been previously reported.

### Stereotypic and repetitive behavior

**Fig 3:**
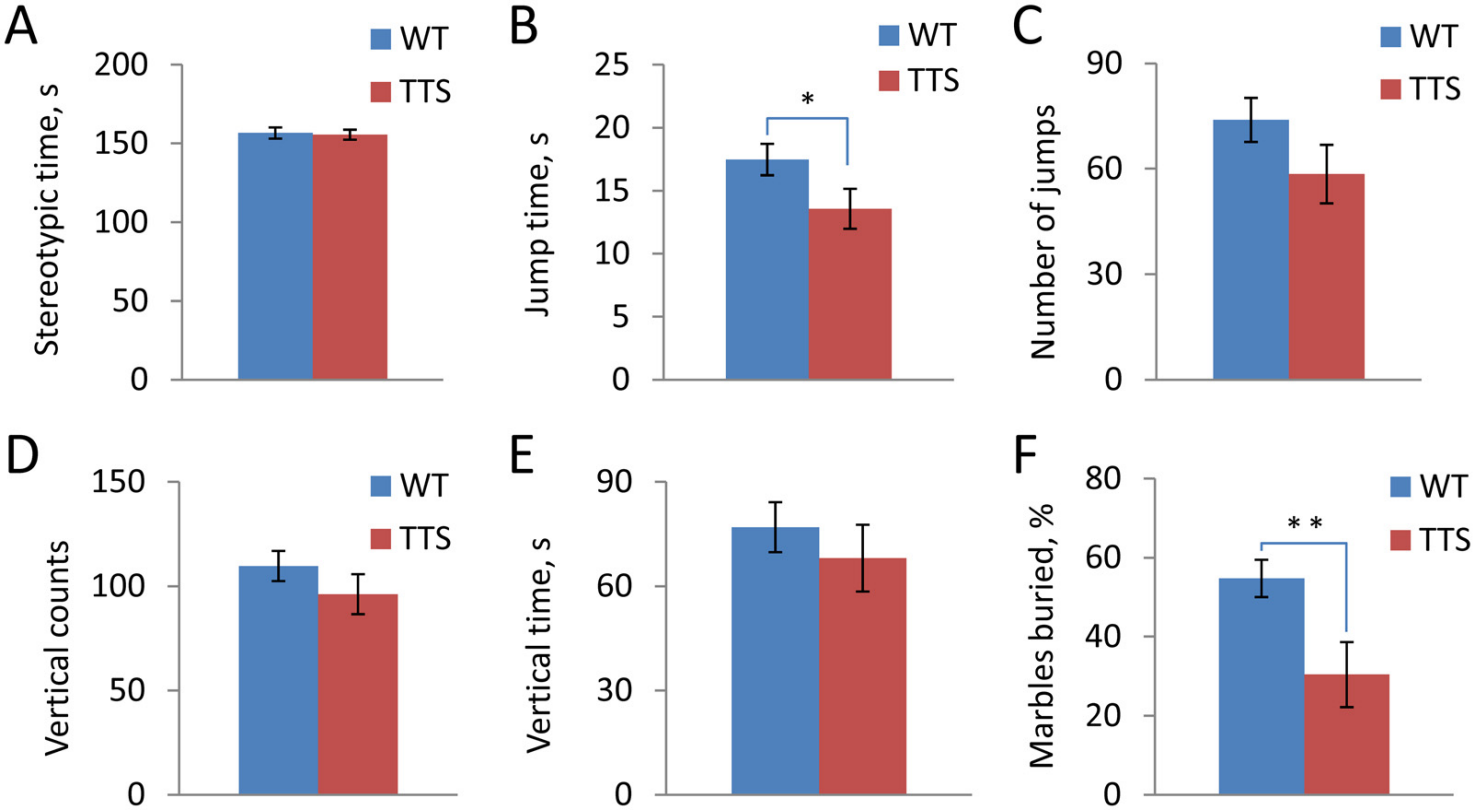
Stereotypic and repetitive behaviors. A: Time spent on stereotypic movements; B: Jumping time; C: The number of jumps; D: Vertical counts; E: Vertical time; F: Percentage of buried glass marbles. Mean ± SEM. Number of tested animals WT = 22; TTS = 12. *p < 0.05 and **p < 0.01, significantly different from WT mice.

Behavioral changes in DS also include increased rates of stereotypic motor movements and repetitive behaviors [69]. To test whether or not such behavioral features are present in TTS mice, we first analyzed repetitive and related behaviors in the ‘activity box’. The time spent on stereotypic movements during a 10-min test in activity box was not different (t(31) = 0.22, p = 0.41), while jumping time was reduced (t(24) = 2.0, p = 0.028) in the TTS vs. WT mice (Fig 3A and 3B respectively). There was no difference in the number of jumps (t(24) = 1.5, p = 0.07), vertical counts (t(23) = 1.2, p = 0.13), and vertical time (t(18) = 1.2, 0.12) in the TTS vs. WT mice (Fig 3C–3E). These data are evidence that stereotypic and repetitive behaviors, as assessed in this study, are not increased in TTS mice.

Obsessive-compulsive behavior of TTS mice was next examined using the glass marble test [70,71]. In this test, the number of glass marbles buried during the testing period is measured, with an increase in the number taken as a positive measure of obsessive-compulsive behavior. The number of glass marbles buried by TTS mice was significantly fewer than in WT mice (t(19) = 2.7, p = 0.008) (Fig 3F). Thus, this test provided no evidence for increased behaviors of this type in the TTS mice.

We conclude that repetitive and stereotypic behavior is not increased, but rather decreased in TTS vs. WT mice. The findings also point to differences with other models examined (Table 1).

### Working memory

**Fig 4:**
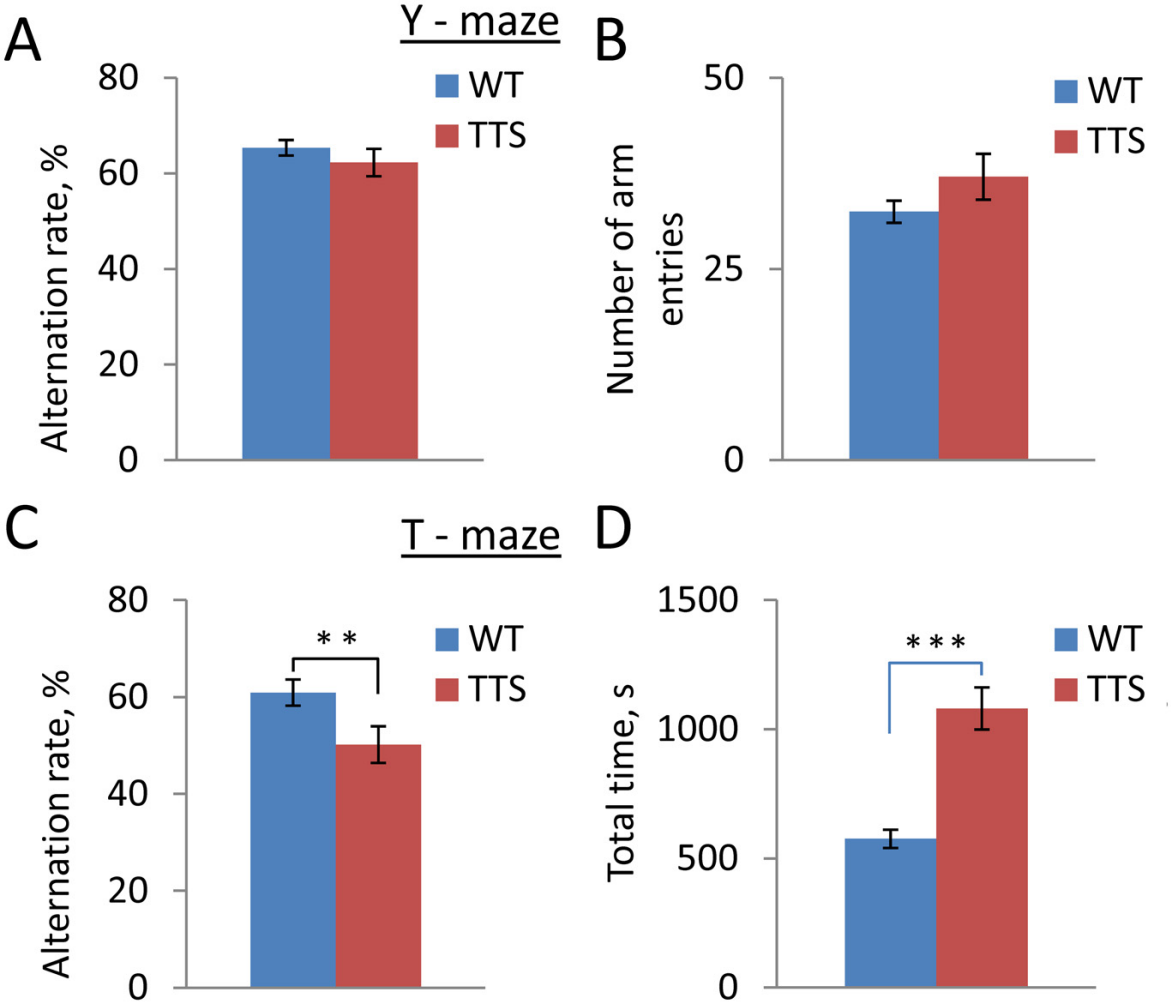
Working memory. A: Alternation rate in Y-maze; B: Number of arm entries in Y-maze; C: Alternation rate in T-maze; D: Averaged time spent on T-maze test. Mean ± SEM. Number of tested animals WT = 22; TTS = 12. **p < 0.01 and ***p<0.001, significantly different from WT mice.

Deficiency of working memory is one of the most significant consequences of genetic alterations contributing to cognitive impairment in DS [72,73]. Because the outcome of the working memory testing could depend on the test complexity, we used two approaches to examine working memory in TTS mice. First, working memory was estimated in the Y-maze, in which the animals’ movements were not restricted (Fig 4A). Averaged for a 5-min testing period, the alternation rate was not different in the TTS vs. WT groups thus providing no evidence for the working memory impairment (Fig 4A, t(19) = 0.97, p = 0.17). Next, working memory was examined in a more demanding T-maze test with forced choices of the target arms during the memory acquisition. In contrast to the results in the Y-maze, T-maze test showed significantly impaired performance in TTS vs. WT mice. Thus, the average alternation rate of TTS mice was close to a chance level (50%), which was significantly lower than the alternation rate registered in WT controls (Fig 4C, t(21), p = 0.01). Thus, working memory was impaired in the TTS mice in this more challenging experimental paradigm. Comparing TTS to other mouse models, the impairment in T-maze testing is shared with most but not all models of DS (Table 1).

In addition to working memory, testing in both Y-maze and T-maze allows for an independent estimation of spontaneous locomotor activity. The number of arm entries during the Y-maze test was not different in the TTS vs. WT mice (t(16) = 1.4, p = 0.09) (Fig 4B), evidence against a change in locomotor activity in this test. In the T-maze, however, the time required to complete the task was almost twice that for WT mice (t(14) = 5.9, p < 0.001) (Fig 4D). This difference can be partly explained by slower movements of TTS vs. WT animals.

Finally, the numbers of pellets during the tests were counted to assess the levels of anxiety in the TTS and WT mice. In the 5-min Y-maze test, the numbers of pellets were in WT: 0.9 ± 0.28; TTS: 0.2 ± 0.11, t (27) = 2.4, p = 0.01. In the T-maze, the average numbers of pellets left per minute were in WT: 0.16 ± 0.04; TTS: 0.15 ± 0.03, t (30) = 0.26, p = 0.40. These data are evidence that the anxiety levels were not increased and, possibly, were even reduced in the TTS vs. WT mice. Thus, difference in the anxiety levels could not account for the deficits in working memory observed in the TTS mice.

### Long-term memory

**Fig 5:**
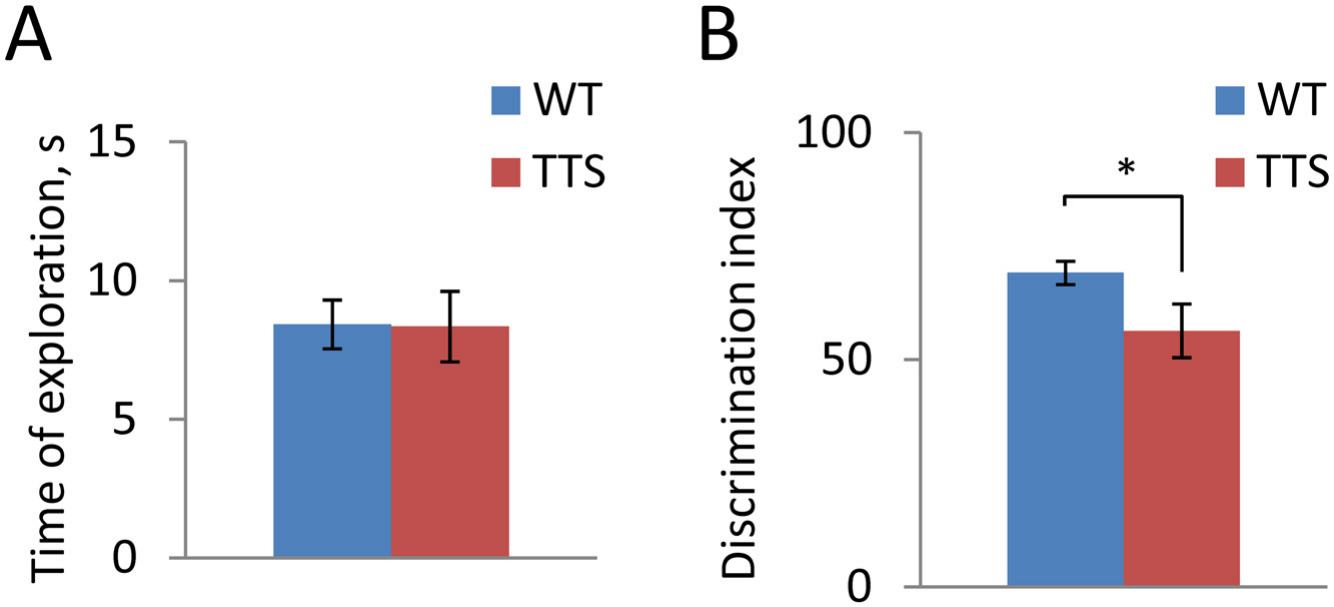
Novel object recognition. A: The time spent on investigation of the objects during the testing phase of the experiment; B: Discrimination index. Mean ± SEM; *p < 0.05, significantly different from WT mice.

Deficiency of long-term hippocampus-dependent memory is characteristic of people with DS [74,75]. To assess long-term memory in TTS mice, we used novel object recognition test with a retention period of 24 h. Previously this test showed a reduction of long-term memory in Ts65Dn mice and other genetic models of DS (Table 1). During the acquisition phase, littermate TTS and WT control mice spent on average an equal amount of time investigating objects suggesting no difference in curiosity between TTS vs. WT mice (WT: 25.2 ± 1.5, ranged between 16–42 s; TTS: 27.6 ± 2.5, ranged between 18–42 s; t(19) = 0.85, p = 0.41). Time of objects exploration was also similar in TTS and WT during the testing phase (Fig 5A, t(18) = 0.05, p = 0.48) However, the discrimination index was significantly reduced in the TTS vs. WT mice (Fig 5B, t(12) = 2.1, p = 0.032) pointing to a decreased ability to detect the novel object. In fact, the performance of TTS mice was not statistically different from chance level (t(10) = 1.13, p = 0.29). Thus, similar to DS, long-term object recognition memory is deficient in the TTS mice as well as in some but not all other models of DS (Table 1).

### Synaptic plasticity

**Fig 6:**
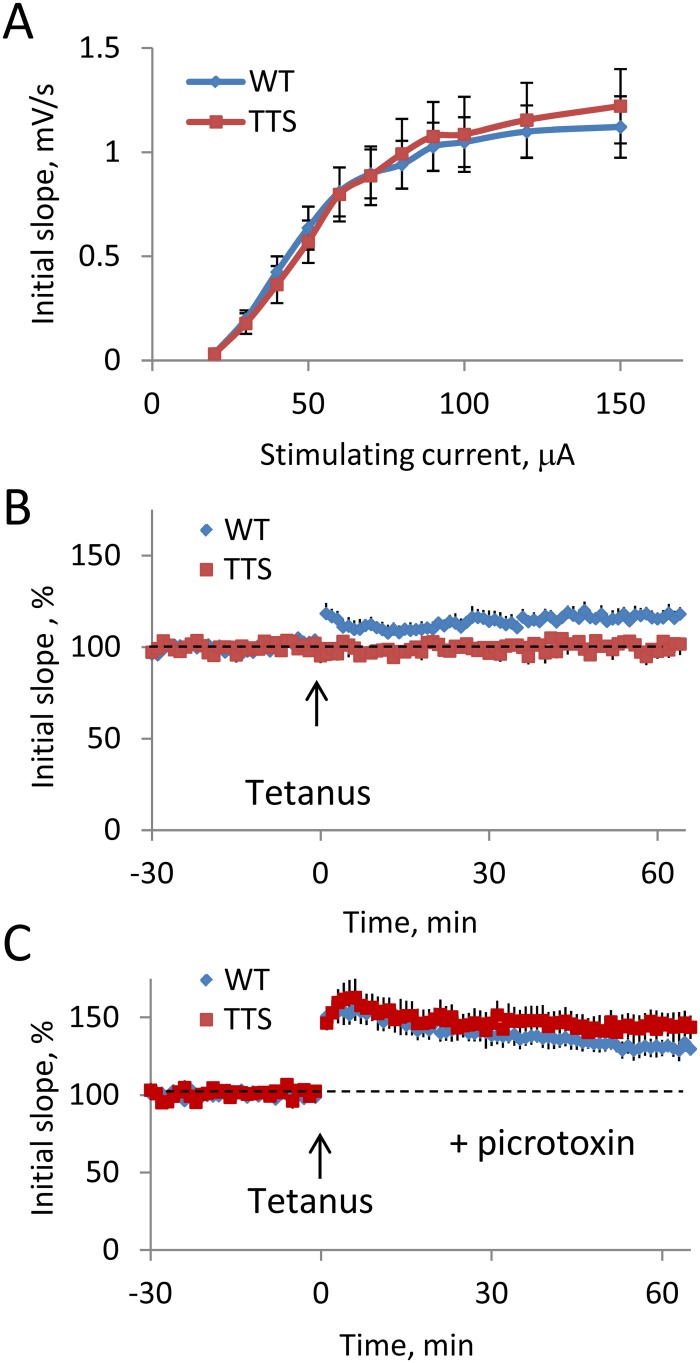
Synaptic efficiency and plasticity in the dentate gyrus. A: Input-output relationship in the DG. Evoked responses were similar in MML of WT and TTS DG (blue and red markers respectively). B: Time course of the averaged initial slope of field EPSPs during the experiment. Tetanization (arrow) evoked stable LTP in WT, but not in TTS mice (blue and red markers respectively). The results are mean ± SEM. The number of slices/mice examined: WT = 9/4; TTS = 8/4. C: Suppression of inhibition by picrotoxin allowed for induction of normal LTP in the TTS slices. The results are mean ± SEM. The number of slices/mice examined: WT = 6/3; TTS = 7/3.

Changes in long-term hippocampus-mediated memory in TTS mice suggested that hippocampal synaptic plasticity may be impaired. Such correlation between impaired long-term memory and decreased hippocampal synaptic plasticity has been previously detected in a number of other mouse genetic models of DS (Table 1). Baseline synaptic efficiency and synaptic plasticity were examined in the DG medial molecular layer in hippocampal slices of TTS and WT mice. Input-output characteristics showed no difference in the baseline efficiency of excitatory neurotransmission in TTS vs. WT slices (Fig 6A). In contrast, tetanizations readily induced LTP in WT, but not in TTS slices (Fig 6B). Averaged for the period 15–60 min responses were significantly greater in the WT vs. TTS slices (Fig 6B, t(13) = 3.7, p = 0.001). The difference between WT and TTS slices was observed immediately after the tetanus (t(15) = 2.9, p = 0.006 for the period 2–14 min) suggesting that induction rather than maintenance of LTP was affected in the TTS mice. To probe whether deficient LTP implicated excessive efficiency of inhibitory neurotransmission, as was observed in other models of DS (Table 1), we measured levels of LTP after suppression of the GABAA receptor-mediated inhibition with picrotoxin (100 μM). Suppression of inhibition allowed for induction of seemingly normal LTP in TTS slices (t (12) = 0.35, p = 0.36 for the period 2–14 min, and t (11) = 0.93, p = 0.19 for the period 15–60 min after the tetanus) (Fig 6C). Thus, deficient LTP in the DG was restored by suppressing of inhibition.

## Discussion

DS phenotypes arise from changes in the dose of genes and/or regulatory sequences on HSA21 [1,3,76,77,78,79]. Here we characterized behavioral and physiological phenotypes in TTS mice, a model of DS in which all known mouse genes orthologous to HSA21 are present in three copies. TTS mice replicate several most important DS phenotypes and show robust differences with respect to WT mice in body weight, locomotor activity, working memory, long term memory, and synaptic plasticity. Importantly, they share some, but not all phenotypes with other segmental trisomy models of DS.

### Mouse genetic models

Mouse genetic models are widely used to study the neurobiology of DS and genotype-phenotype relationship in DS. Previous studies have made important contributions to define the genes and underlying mechanisms responsible for cognitive challenges and other phenotypes. Similar to other genetic disorders, the approach that informs the process of deciphering DS-relevant insights follows the pattern: 1) define an important human disease phenotype; 2) determine whether or not the phenotype is present in the model; 3) carry out studies to define candidate genes; 4) provide evidence to confirm the identity of the gene(s) responsible; and 5) explore the mechanism(s) induced. Each step provides challenges. In DS, the most difficult part is deciphering of the genotype-phenotype relationship(s). Indeed, abundance of the triplicated genes and the corresponding phenotypes greatly complicates the analysis. The availability of models harboring different genetic segments syntenic to HSA21 that can be shown to demonstrate conservation, or lack thereof, of the phenotype of interest, has and will continue to facilitate mapping phenotypes to genes and mechanisms.

An important limitation to progress is that the collection of available models falls short of the ideal for representing mouse homologues of HSA21. Thus, full trisomy 16 (Ts16) mice containing three copies of the entire Mmu16 have many genes not shared with HSA21 and can only be studied prenatally [80,81,82,83]. The most studied model, the Ts65Dn mouse, carries an extra copy of Mmu16 segment with ~100 of HSA21 gene orthologs. It was the first model to allow for phenotyping beyond the fetal period [54,63] and has proven extraordinarily useful. Unfortunately, this model also harbors three copies of about 36 human gene orthologs not present on HSA21 [84,85]. Although Ts65Dn mice show many features of DS, triplication of non-DS genes could, in principle, result in phenotypes not attributable to increased dose of genes on HSA21. More recent models that harbor smaller genetic segments from mouse chromosomes syntenic to HSA21 are the Ts1Cje, Ts1Rhr, and Ts1Yah mice. These models contain respectively 69, 29, and 12 HSA21 orthologs [16,17,18,84,85] which we estimate represent approximately 39.4%, 16.6%, and 6.85% of the total number of known gene orthologs triplicated in DS. These models are useful for exploring phenotypes created only by genes present in these smaller segments.

Another genetic model of DS is the Tc1 mouse, which carries a large part of HSA21 [15,65]. This mouse initially appeared to be ideal for studies linking DS phenotypes to genes. However, recent analysis revealed a number of notable changes in structure of HSA21 in Tc1 mice including at least one large deletion, six duplications, and more than 25 *de novo* structural rearrangements [86]. Moreover, only a subset of neural cells (~50%) contains this human genetic material. An additional concern, and one of uncertain significance, is that interactions between the proteins encoded by human and murine orthologs may differ. This could result in changes that would not emerge in cells harboring only mouse or human genes.

Finally, a number of DS models contain increased or reduced dosage of single genes or small genomic regions implicated in DS [87,88,89,90,91,92,93]. These models have proven useful for dissecting the roles of individual genes in DS phenotypes. However, the relevance to DS of phenotypes observed in ‘single-gene’ models must be examined for both sufficiency and necessity in the context of more complete genetic models.

Recently, each of the mouse syntenic chromosomal segments on Mmu10, Mmu16, and Mmu17 homologous to HSA21 has become available for genetic engineering. Mice harboring an extra copy of individual segments or their combinations can now be produced and experimentally studied [43]. The most complete is the TTS mouse which contains an extra copy of all three mouse syntenic regions homologous to HSA21. However, even for this most advanced DS model one must note that the mouse and human genomes, though homologous for genes on HSA21, are not identical [94]. Our studies in the TTS mice focused on documenting the presence or absence of behavioral and physiological phenotypes that correspond to those present in DS and other mouse genetic models of DS.

### Locomotion and repetitive motor movements

People with DS are characterized by notable perceptive-motor slowness [95,96], stereotypic repetitive behavior [69,97], and high prevalence for the hyperkinetic and attention deficit hyperactivity disorders [98]. In animal models, such alterations might be evidenced, at least in part, by changes in spontaneous locomotor activity. Indeed, earlier studies in Ts65Dn mice showed increased spontaneous locomotor activity at all ages studied [25,26]. Locomotor activity was apparently increased in Tc1 mice [59], as well as in some other models of DS [92,99]. Herein we observed mildly increased locomotion in TTS mice, which was most evident in increased ambulatory time. On the other hand, averaged velocity of movement was decreased in the TTS mice (Table 1). Consistent with decreased velocity, TTS mice required considerably longer time to complete the task in the T-maze. Decreased velocity of movement, though statistically highly significant in the TTS mouse, has not been observed in any other model of DS. On the contrary, an increased velocity and a corresponding increase of ambulatory distance were detected in both the Ts65Dn [25] and Tc1 [59] mice. If the discrepancy is confirmed in the TTS and other models using more exhaustive measures of locomotion, it would point to ambulatory velocity as a DS-relevant phenotype whose genetic an mechanistic basis should be explored. In this respect, it is interesting that earlier studies of Ts1Cje mice reported a tendency for reduced locomotor activity [17,64], while Ts1Rhr mice exhibited no such changes [45]. This suggests that genes affecting locomotor activity may be present in the segments that differ between these models.

In future studies, it will be important to explore the underlying mechanisms and nervous system loci responsible for the changes in locomotor velocity as well as for other motor manifestations, such as reduced swimming velocity and grip strength, in TTS mice [43]. Interestingly, the decrease in swimming velocity was conserved in Dp(16)1Yey/+, but not in Dp(10)1Yey/+ or Dp(17)1Yey/+ mice, suggesting that the genes present on Mmu16 may contribute to this phenotype (Table 1). In future studies it will be important to examine how other motor phenotypes are conserved in each of these mice. We conclude that the TTS mouse demonstrates a number of motor phenotypes that are similar to those in DS [100], but different from the corresponding phenotypes observed in other mouse DS models.

### Obsessive-compulsive behavior and anxiety

An increased rate of obsessive-compulsive behavior is characteristic of children with DS [101]. In adult individuals, however, the prevalence rate for obsessive-compulsive disorder is not different in DS (0.8–4.5%) [102,103] vs. general population (1–4%) [104,105]. Using the glass marble test, a standard approach for estimating obsessive-compulsive behavior [71], we observed no evidence for increases in TTS vs. WT mice. Indeed, TTS mice buried a smaller number of glass marbles during the test, which could be interpreted as a reduction of obsessive-compulsive symptoms. Complicating the analysis is that locomotor velocity is reduced in TTS mice. Because this test is sensitive to levels of locomotor activity [106,107], smaller number of buried marbles in the TTS mice can be explained, in part, by their relative slowness. A contribution of reduced locomotor activity and strength of TTS mice to our findings must be addressed.

Anxiety is present in some people with DS, but the frequency of this condition is not marked. Indeed, only about 5% of people with DS age 50 years or less, and about 9% at the age greater than 50 years, were diagnosed with anxiety [108], rates comparable to those in the general population [109]. The lack of excessive anxiety in TTS mice was supported by a number of our measurements. As such, the data are consistent with studies in most of the models in which anxiety has been examined (Table 1).

### Working and long-term memory

Deficiency of working memory is an important characteristic of DS that contributes to cognitive inability [110,111]. Several genetic models of DS, including the Ts65Dn, Ts1Cje, Ts1Rhr, and Ts1Yah mice, demonstrate deficiencies in working memory [3,16,25,26,37,112] (Table 1). Thus, changes in working memory are represented across models with genomes that differ greatly in size and composition. Herein we observed in adult TTS mice a deficit in working memory in the T-maze, but not in the Y-maze. One explanation for the apparent discrepancy is that the T-maze test is more challenging. Indeed, in the T-maze the mouse was kept idle for at least 12 s between the acquisition and testing trials, while in Y-maze the intervals between the trials were not regulated and, hence, were generally shorter. Deficiency of working memory in the T-maze agrees with our previous findings of reduced working memory of TTS mice in Morris water maze (Table 1). Interestingly, deficiency of working memory was observed in a number of different DS models (Table 1), pointing to the possibility that genes on more than one segment may contribute.

Perhaps, the most salient features of DS models are represented by deficient hippocampus-dependent memory and long-term synaptic plasticity, both of which provide the basis for understanding the deficient cognition in DS [26,113,114]. The majority of genetic models of DS showed decreased performance in novel object recognition and other tests for long-term hippocampus-dependent memory, as well as decreased LTP in the CA1 and DG.

Reduced performance in the novel object recognition test with the retention period of 24–48 h was observed in Ts65Dn [23,30,38,66], Ts1Yah [16], and Ts1Rhr [45] mice. In contrast, Tc1 [65] and Ts1Cje [67] mice showed no memory changes when examined in this test. Herein we observed deficient performance of TTS mice in the novel object recognition test. The results complement our early findings of reduced contextual memory of the TTS mice in the contextual fear conditioning, as well as reduced performance in the Morris water maze [42]. Importantly, the deficits in these tests were conserved in the Dp(16)1Yey/+, but not in Dp(10)1Yey/+ or Dp(17)1Yey/+ mice (Table 1), pointing to the homologous segment on Mmu16 as necessary.

Finally, we observed that LTP in DG MML was deficient in the TTS mice. Similar changes in LTP were previously demonstrated in Ts65Dn [26,30,38] and all the models in which LTP was tested in DG [45,65,115]. Importantly, the conservation of decreased LTP in the DG was accompanied by evidence of normalization using picrotoxin to block GABAA receptors. Studies in the CA1 region of the TTS mice also showed deficient LTP [42]; this was conserved in the Dp(16)1Yey/+ mouse [42]. Remarkably, increased LTP was demonstrated in the Dp(17)1Yey/+ [42] as well as in the Ts1Yah mouse [16], pointing to the possibility that genes on different segments may interact to produce relevant physiological phenotypes.

An intriguing observation of this study is a relatively mild expression of phenotypes in the TTS mice. Indeed, many of the observed phenotypes were less pronounced in TTS vs. Ts65Dn mice. The phenotypic variations between TTS and Ts65Dn mice could be affected by several factors. First, in contrast to Ts65Dn mice, in which additional genetic material is present in the form of a discrete ‘mini-chromosome’, in the TTS mice additional genetic material homologous to HSA21 is contained within the corresponding mouse chromosomes (Mmu10, Mmu16, and Mmu17). Genomic neighborhood strongly affects the regulation of gene expression (e.g. [116]). Thereby, different organization of the additional genetic material could affect neuronal properties resulting in the exacerbation of the abnormal phenotypes in Ts65Dn mice. Second, the triplication of the Mmu17 centromeric region which contains copies of about 60 non-DS Mmu17 genes [84] could also contribute to phenotypes in Ts65Dn mice. Third, Ts65Dn and TTS mice contain an extra copy of 57.1% and 100% of the HSA21 gene orthologs, respectively. The contribution of these additional HSA21 gene orthologs may modify the phenotypic changes in the TTS mice vs. those registered in Ts65Dn. Finally, TTS and Ts65Dn models are on different strain backgrounds. Genetic background differences affected DS phenotypes in the Ts1Rhr mice [117]. Note that, while no change in hippocampal LTP was observed in Ts1Rhr mice inbred on the C57BL/6J background [18,21,118], this phenotype was present in Ts1Rhr mice inbred on B6EiC3Sn/J [45]. The role of the genetic background in expression of DS phenotypes in mouse models is evident and should be further examined in future studies. The stage is now set to more effectively discover and decipher DS-relevant phenotypes in genetic models of DS, and the TTS mouse can serve to simplify and focus these studies.

## Conclusions

In conclusion, TTS mice exhibited changes in body weight, locomotion and general fitness, deficient working memory and long-term memory, and reduced synaptic plasticity as measured by induction of LTP in the dentate gyrus. Thus many, but not all of the phenotypes described in previous models of DS were detected in TTS mice. This unexpected finding points to complexity of the genotype-phenotype relationship in DS. Indeed, it is likely that some of the phenotypes observed in previous, genetically less complete DS models, may arise from unopposed action of genes, whose effects are counterbalanced in the TTS mice by action of other triplicated genes. A question thus arises: Which of the DS models should be considered as more adequate—models with stronger phenotypes (e.g., Ts65Dn mice) or models most closely replicating genetic changes observed in DS (e.g., TTS mice)? We view the genetic correctness as the most important feature for a genetic model. As such, we point to TTS mice as a new standard by which to compare and guide studies defining genotype-phenotype relationships in DS.

## Supporting Information

S1 Table Correlation coefficients between the measured behavioral parameters and the animal’s age. Neither of the correlations reached the level of significance suggesting no impact of the animal’s age on the measured behavioral parameters.
(DOCX)
