# Supplementary material for: Down Syndrome Cognitive Phenotypes Modeled in Mice Trisomic for All HSA 21 Homologues
Source: PLoS One. 2015 Jul 31;10(7):e0134861. doi: 10.1371/journal.pone.0134861 (PMC4521889; doi:10.1371/journal.pone.0134861)
Supplement: S1 Table — Neither of the correlations reached the level of significance suggesting no impact of the animal’s age on the measured behavioral parameters. (DOCX) [file pone.0134861.s001.docx]

| **Supplemental Table S1. Correlation coefficients between the measured behavioral parameters and the animal’s age** | | |
| --- | --- | --- |
| Behavioral parameters | 2N | Ts65Dn |
|  | R crit (0.05) = 0.44 | R crit (0.05) = 0.58 |
| Locomotor Activity |  |  |
| Ambulatory Distance | 0.00 | 0.48 |
| Ambulatory Time | -0.03 | 0.46 |
| Resting Time | 0.04 | -0.39 |
| Velocity | 0.18 | 0.24 |
| Stereotypic Time | 0.00 | 0.13 |
| Number of Jumps | 0.03 | -0.04 |
| Jump Time | -0.19 | -0.14 |
|  |  |  |
| Other Tests |  |  |
| NOR Exploration time Day 1 (Acquisition) | 0.29 | 0.08 |
| NOR Exploration time Day 2 (Testing) | 0.00 | 0.47 |
| NOR Discrimination Index | -0.38 | 0.27 |
| Y-maze Alternation Rate | 0.08 | 0.03 |
| Y-maze Arm Entries | -0.04 | 0.27 |
| T-maze Alternation Rate | 0.20 | -0.15 |
| T-maze Total time | -0.04 | 0.35 |
| Marble test, Marbles Buried | 0.25 | -0.38 |

Neither of the correlations reached the level of significance suggesting no impact of the animal’s age on the measured behavioral parameters.
